# Chronic Paronychia and Onychomadesis in Pemphigus Vegetans: An Unusual Presentation in a Rare Autoimmune Disease

**DOI:** 10.1155/2018/5980937

**Published:** 2018-01-11

**Authors:** Thanisorn Sukakul, Supenya Varothai

**Affiliations:** Department of Dermatology, Faculty of Medicine Siriraj Hospital, Mahidol University, Bangkok, Thailand

## Abstract

Pemphigus vegetans is a rare variant of deep acantholytic pemphigus which usually presents with vesiculobullous rash and vegetative plaques on the folds. We report a case of pemphigus vegetans patient who presented with rashes on tips of fingers and toes resembling paronychia and onychomadesis that misled the diagnosis for months. The final diagnosis of Hallopeau-type pemphigus vegetans was made based on histopathology and direct immunofluorescence studies. Interestingly, not only the clinical presentation was atypical, but blood tests for anti-desmoglein 1 and 3 antibodies by ELISA technique were also negative. Thus, the rare unusual manifestation of pemphigus vegetans in this patient may associate with different autoantibodies to desmosomal proteins compared with those of classic pemphigus patients.

## 1. Introduction

Pemphigus vegetans (Pveg) is a rare variant of deep acantholytic pemphigus which is mostly found in middle-age women patients. The typical presentations are deep red irregularly or polycyclic mammillated plaques with macerated epithelial collarette border on intertriginous area, evolving into vegetative plaques [1]. Cerebriform tongue is a sign of mucosal involvement commonly found in patients [2]. Two types of Pveg were described: (1) Neumann type which forms vesicles and bullae before turning into dry, hyperkeratotic plaques with fissure and (2) Hallopeau type which forms centrifugal expansion vegetative lesions without vesicles or bullae formation. The second type is less aggressive and localized [1]. The diagnosis is made on clinical presentations, pathohistology and immunofluorescence studies, and/or circulating autoantibodies recognition using immunoblot and ELISA analysis [3]. We hereby presented a case of Pveg with atypical presentation of chronic paronychia and onychomadesis.

## 2. Case Presentation

**Figure 1:**
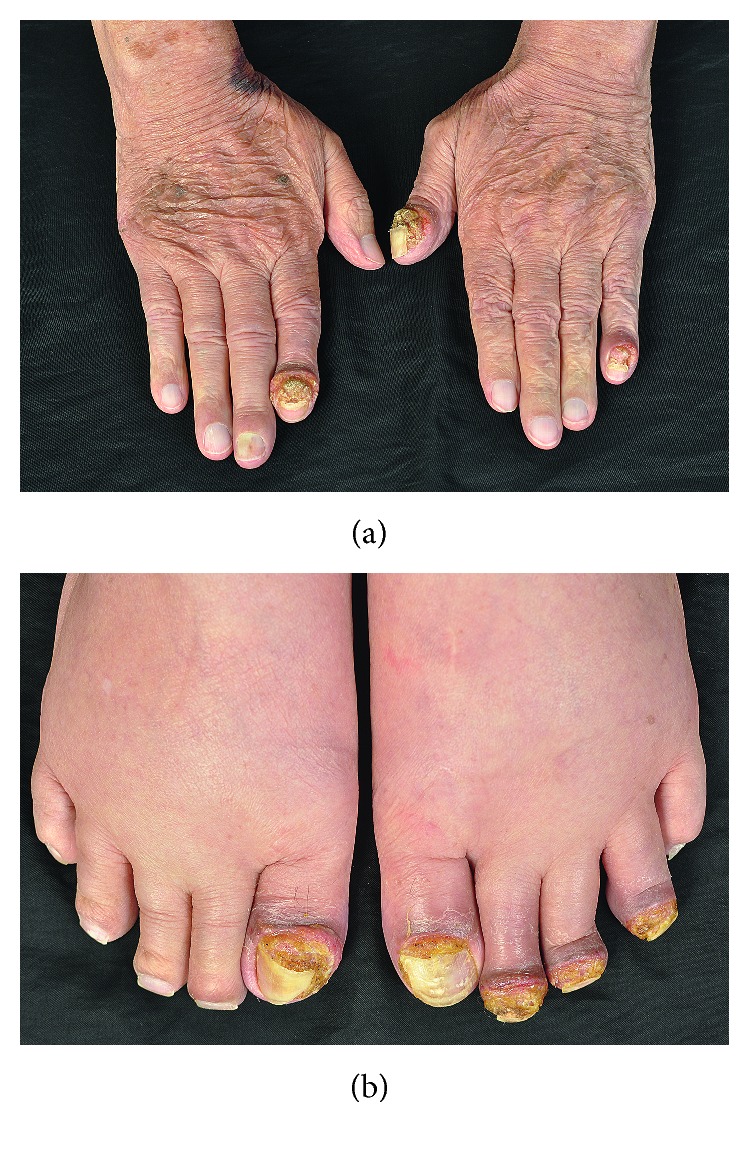
Clinical presentation of a 77-year-old patient with paronychia-like lesions and onychomadesis at tips of fingers and toes.

A 77-year-old male with type 2 diabetes mellitus and hypertension presented with itchy scaly erythematous patches and plaques on his face and trunk for 5 months. He was initially diagnosed as having subacute eczema. The patient had no mucosal involvement or systemic symptoms and denied history of over-the-counter or herbal drug use. The rashes mostly subsided after treatment with topical corticosteroids (mometasone furoate 0.1% cream) for a few months. Four months after treatment, he developed swelling of proximal nail folds and brittle nails of many fingers and toes resembling chronic paronychia, followed by nail detachment and crusted plaques on proximal and lateral nail folds (Figure 1). The differential diagnosis at that time included acrodermatitis continua of Hallopeau and acrodermatitis paraneoplastica of Bazex. Few weeks later, a scaly erythematous patch on left groin progressed into an infiltrative mass without serum oozing, pus, or vesicles. Physical examination of other systems was unremarkable. Complete blood count, liver and renal function tests, chest X-ray, and computerized tomography of the chest and whole abdomen revealed normal.

**Figure 2:**
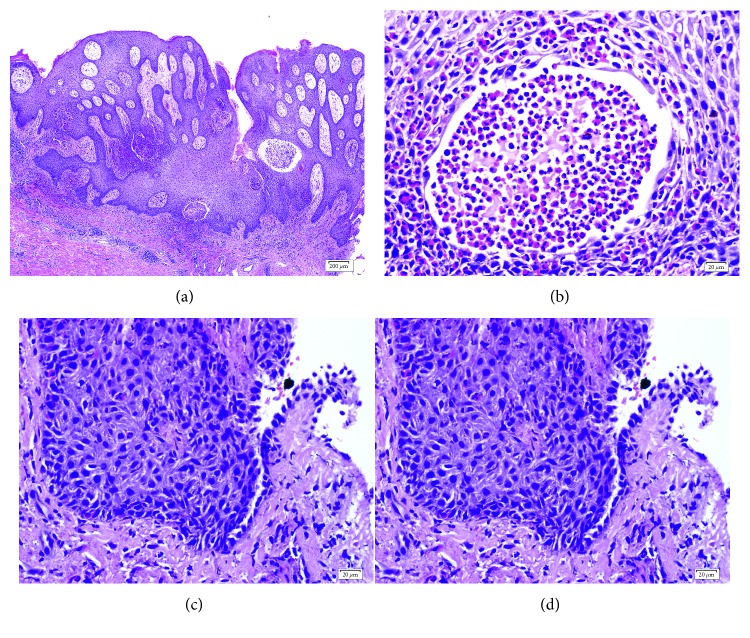
Histopathology study (hematoxylin and eosin staining) demonstrated pseudoepitheliomatous hyperplasia of epidermis with eosinophilic spongiosis (a), intraepidermal eosinophilic microabscess with few acantholytic cells (b), and focal area of suprabasal separation (c).

**Figure 3:**
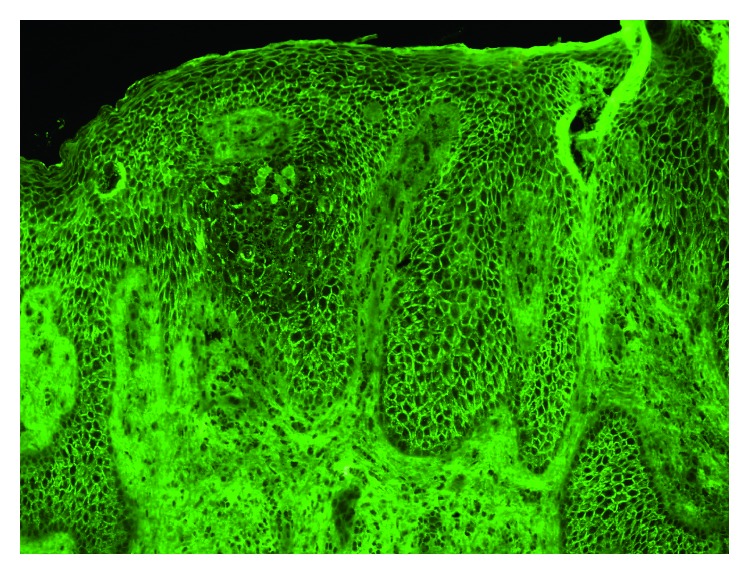
Direct immunofluorescence study showed positive IgG at the intercellular space of the epidermis.

The skin biopsy from the lesion on the left groin was done for diagnosis. Histopathological study showed pseudoepitheliomatous hyperplasia of epidermis with eosinophilic spongiosis and intraepidermal eosinophilic microabscess. Few acantholytic cells and focal area of suprabasal separation were observed (Figure 2). Direct immunofluorescence (DIF) study showed positive IgG and C3 at intercellular space of epidermis (Figure 3). The diagnosis of Pveg was made. However, blood test for anti-desmoglein (Dsg) 1 and 3 antibodies (ELISA) was negative. Systemic oral prednisolone (1 mg/kg/day) was prescribed for the first few months, and then the dose was tailed off and discontinued after 6 months of treatment. The lesions were improved and remained clear with topical corticosteroids cream (clobetasol propionate 0.05% cream) application.

## 3. Discussion

The initial clinical presentation of this patient misled the diagnosis to eczema and acrodermatitis in psoriasis as mentioned. Absence of vesicles, bullae, and typical vegetative plaque on the folds and oral mucosa retarded the diagnosis of Pveg at a time. Finally, diagnosis of Pveg was confirmed by histopathological and DIF studies. Hallopeau-type Pveg was preferred because of the localization of thickened expansion plaques without bullae formation and gradual response to systemic steroids. Although many case reports of various presentations of Pveg were published [4–9], only few reports mentioned lesions on tips of fingers and toes and/or nail involvement [10–13]. Furthermore, most of the patients in previous reports had other clinical presentations which suggested that the diagnosis of Pveg and the blood test for autoantibodies to desmosomal proteins were not done. Recently, some authors reported Pveg in association with other diseases, drugs, and malignancy [14–20]. However, other physical examination and investigations in this patient were found to be normal.

Interestingly, blood for anti-Dsg1 and anti-Dsg3 antibodies (ELISA) in this patient were negative. Although blood for anti-Dsg (ELISA) is an effective tool for the diagnosis of pemphigus vulgaris and pemphigus foliaceus with more than 97 percent of sensitivity and specificity, the data are still limited for Pveg [21]. For nonclassical pemphigus (paraneoplastic pemphigus, pemphigus herpetiformis, and Pveg), the antibodies to desmosomal proteins may be different from classical pemphigus including antibodies to desmocollins (Dsc) and periplakin [22–25]. Ishii et al. found that considerable numbers of Pveg sera reacted strongly with Dsc and might not react with Dsg [26]. Mergler et al. also noted that anti-Dsg1 and anti-Dsg3 detection does not correlate with the skin and/or mucosal involvement [27]. These may further help explaining the difference in clinical appearance between classic pemphigus and typical and atypical presentation of Pveg. Blood test for anti-Dsc antibodies of this patient was not performed due to limited laboratory facilities in our institute.

In conclusion, we report a case of Pveg that presented with paronychia-like lesions and onychomadesis with positive DIF study but negative anti-Dsg1 and anti-Dsg3 antibodies in blood. We highlight the atypical presentation which may be associated with the difference of autoantibodies to desmosomal proteins in pemphigus patients. Further investigation to find out the autoantibodies should be done to define the abstruse pathogenesis of this rare variant of disease.
